# Circulating Ang-2 mRNA Expression Levels: Looking Ahead to a New Prognostic Factor for NSCLC

**DOI:** 10.1371/journal.pone.0090009

**Published:** 2014-02-28

**Authors:** Ana L. Coelho, António Araújo, Mónica Gomes, Raquel Catarino, Agostinho Marques, Rui Medeiros

**Affiliations:** 1 Molecular Oncology Group, Portuguese Institute of Oncology Porto, Porto, Portugal; 2 Faculty of Medicine, University of Porto, Porto, Portugal; 3 Medical Oncology Department, Centro Hospitalar de Entre o Douro e Vouga, Santa Maria da Feira, Portugal; 4 Abel Salazar Institute for the Biomedical Sciences, University of Porto, Porto, Portugal; 5 Pulmonology Department, Centro Hospitalar de S. João, Porto, Portugal; 6 Research Department, Portuguese League Against Cancer (NRNorte), Porto, Portugal; The Chinese University of Hong Kong, Hong Kong

## Abstract

Non-small cell lung cancer (NSCLC) is the most common cancer and the leading cause of death from cancer worldwide. Antiangiogenic strategies directed towards tumor stroma are becoming gold standard in NSCLC treatment and researchers have been searching for biomarkers to identify patients for whom therapy with antiangiogenic inhibitors may be most beneficial and the importance of these as prognostic factors in NSCLC. The purpose of this study was to evaluate the prognostic value of circulating Ang-2 mRNA levels prior to treatment in NSCLC patients. The mRNA levels were determined by quantitative real-time PCR in the peripheral blood of 92 NSCLC patients. Our results demonstrate that patients with high circulating Ang-2 mRNA levels have diminished overall survival when compared to those with low mRNA levels (20.3 months vs 34.3 months, respectively; Log Rank Test, p = 0.016), when considering all NSCLC stages and this difference is even bigger when considering only patients with stage IV (15.9 months vs 31.3 months, respectively; Log Rank Test, p = 0.036). Moreover, circulating Ang-2 mRNA levels independently determine overall survival, and the concordance (c) index analysis showed that the definition of a nomogram that contains information regarding tumor stage, patients' smoking status and circulating Ang-2 mRNA levels present an increased capacity to predict overall survival in NSCLC patients (c-index 0.798). These results suggest that this nomogram could serve as a unique and practical tool to determine prognosis in NSCLC, not relying on the availability of adequate surgical or biopsy specimens of NSCLC. Attending to our results, the circulating Ang-2 mRNA levels should also be included in the design of preclinical studies and clinical trials involving antiangiogenic drugs targeting Ang-2, to guide adequate patient stratification and dose selection and increasing the likelihood of benefit to a level that is acceptable to patients and clinicians.

## Introduction

Non-small-cell lung cancer is the most frequent type of lung cancer and the most common cause of death from cancer [1]. In 2010, the number of deaths from lung cancer worldwide was 1·5 million, representing 19% of all cancer deaths that year. Most lung cancers (∼80%) are non-small-cell lung cancers (NSCLC) and of these patients, more than 65% present with locally advanced or metastatic disease [2].

Solid tumors, including NSCLC, require angiogenesis—the formation of new blood vessels from existing vessels—for survival, growth, and metastasis. These new tumor vessels are structurally and functionally abnormal. They develop by sprouting or intussusception from pre-existing vessels and exist in a constantly dynamic state of sprout formation, proliferation, remodeling, or regression [3], [4].

In the last 9 years, antiangiogenic therapy has become part of standard antitumor treatment. However, the clinical efficacy of such therapies is limited, and it appears that the full therapeutic potential of antiangiogenic intervention has not been fully exploited [5].

It's now known that there are various molecular players involved in different mechanisms of vascular growth in solid tumors, and among these, members of the Vascular Endothelial Growth Factor (VEGF) and Angiopoietin (Ang) family have a predominant role [3].

Angiopoietins, the bona fide ligands of Tie-2 receptor, form a family of secreted 70 kDa glycoproteins acting primarily on the vasculature to control blood vessel development and stability. Four distinct angiopoietins have been described: Ang-1, Ang-2, Ang-3 and Ang-4. Angiopoietins bind the second immunoglobulin motif of Tie-2 whereby they activate Tie-2 and, indirectly, Tie-1 in Tie-1/Tie-2 heterodimers [6].

Ang-1 is expressed by pericytes, smooth muscle cells, and fibroblasts and acts in a paracrine manner. In contrast, Ang-2 is expressed by endothelial cells (EC) and stored in the Weibel-Palade bodies from where it can be rapidly released on stimulation to act as an autocrine regulator of EC functions [7].

Ang-1 and Ang-2 have been described to exert opposing functions during vessel development. Ang-1–induced Tie2 activation transduces survival signals and leads to vessel stabilization and maturation. In turn, Ang-2 acts as a vessel destabilizing agent that induces permeability and leads to dissociation of cell-cell contacts in cultured endothelial cells. Genetic experiments have solidly established Ang-2 as an antagonistic Tie2 ligand [7]. Moreover, Ang-2 can have a direct pro-angiogenic Tie-2-independent role by directly binding integrins in Tie2 negative EC [6].

Ang-2 has been implicated in the remodeling of the tumor vasculature in a process resembling its physiological actions [8], [9]. Among the first steps of the angiogenic switch is the co-optive engagement of the pre-existing host vasculature by the growing tumor. This results in EC activation and intense Ang-2 expression, which promotes the dissociation of pericytes from pre-existing vessels and increases vascular permeability, which facilitates the infiltration of proteases, cytokines and angiogenic myeloid cells, and thus, the priming of the vasculature for a robust angiogenic response in the presence of growth factors, such as VEGF-A [6]. Following the angiogenic switch, the Ang–Tie system contributes, in concert with VEGF, to tumor angiogenesis [10].

Ang2 is strongly regulated at the transcriptional level. In fact, almost any form of endothelial cell activation leads to upregulation of Ang2 mRNA. The mRNA induction of Ang2 in tumor endothelium has made Ang2 a very attractive circulating biomarker of angiogenic activation [5].

Some studies have addressed the correlation between Ang-2 expression in tumor tissue and the protein circulating levels and cancer development and metastasis. However, few clinical studies have documented a correlation between this molecule and disease clinical features or prognosis in lung cancer [11]-[15].

To the best of our knowledge, the present study is the first to establish a correlation between circulating Ang-2 mRNA levels and lung cancer prognosis.

## Materials and Methods

### Ethics statement

The study was conducted according to the principles of the Helsinki Declaration. The study was approved by the local ethics committee at the Portuguese Institute of Oncology of Porto (Portugal). All individuals signed a written informed consent prior to the inclusion in the study.

### Study Population

The study included Caucasian patients from the North region of Portugal. The inclusion criteria were histological or cytological confirmed diagnosis of NSCLC, no previous treatment, an Eastern Cooperative Oncology Group (ECOG) performance status≤2 (with 0 indicating that the patient is fully active, 1 that the patient is ambulatory but restricted in strenuous activity, and 2 that the patient is ambulatory and capable of self-care but is unable to work [16]), no prior oncologic disease and available clinical data.

### Circulating mRNA levels quantification

Circulating Ang-2 mRNA levels were analyzed by quantitative real-time PCR (qRT-PCR). Initially, the mRNA was isolated from the cell fraction of peripheral blood samples by TriPure reagent (Roche Applied Science), and after separation of the RNA fraction, the samples were purified using the commercial kit GeneJET RNA Purification Kit (Fermentas). RNA samples were then used as templates for cDNA synthesis, using a High Capacity RNA-to-cDNA Kit (Applied Biosystems), according to the manufacturer's instruction. Finally, qRT-PCR was carried out on a StepOne ^TM^One qPCR equipment, containing 1x Master Mix (Applied Biosystems), with 1x probe (TaqMan Gene Expression Assay with reference number Hs 01048042_m1, Applied Biosystems), cDNA sample, human GUSB (Beta Glucuronidase) and human β-2M (β-2 Microglobulin) endogenous controls (both from Applied Biosystems) according to manufacturer's instructions.

To quantify the amplified transcripts, we used the comparative CT (2^−ΔΔC^T) method [17]. In accordance with the method, the mRNA amounts of the target gene (*Ang-2*) were normalized to two endogenous controls and relatively to a calibrator. We used the housekeeping genes *GUSB* (Applied Biosystems) and *β-2M* (Applied Biosystems) as internal controls and commercial RNA controls as calibrators (Applied Biosystems). ΔΔCT represents the difference between the mean ΔCT value of a patient blood sample and the mean ΔCT value of the calibrator, both calculated after the same PCR run, whereas ΔCT is the difference between the CT of the target gene and the CT of the endogenous reference gene of the same sample. The relative quantitative value was expressed as 2^−ΔΔ^CT. Relative quantification (RQ) based on the Ct (the number of PCR cycles necessary to obtain the threshold signal of fluorescence) values was analyzed using Applied Biosystems StepOne Software v 2.2. All samples were run in duplicate.

### Statistical analysis

Ang-2 mRNA expression levels were considered as categorical variables using the first quartile as cut-off point. We defined that the values under the cut-off point should be included in the low expression group and that all the other cases above the cut-off point constituted the high expression group. Overall Survival (OS) was calculated from the beginning of treatment to death from any cause. Median OS was estimated with the Kaplan-Meier method and compared with a two-sided log-rank test. Multivariate Cox proportion analysis was performed to determine the influence of age, gender, tumor stage, histological type, smoking status and circulating Ang-2 mRNA levels on OS in NSCLC patients. Hazard ratios (HRs) estimated from the Cox analysis were reported as relative risks with corresponding 95% confidence intervals (CIs). The extent of discrimination of the predictive ability was quantified using the Harrel's concordance index (c-index), which estimates the probability of concordance between predicted and observed responses. The interpretation of the C index is similar to the interpretation of the area under a receiver operating curve (ROC) curve. A value of 1.0 indicates that the features of the model perfectly separate patients with different outcomes while a value of 0.5 indicates the features contain prognostic information equal to that obtained by chance alone.

All analyses were performed using Statistical Package for Social Science (SPSS) for Windows version 18 (Chicago, IL). The level of statistical significance was set at 5% (P≤0.05).

## Results

The study included 92 Caucasian individuals from the North region of Portugal, with histopathological diagnosis of NSCLC, with a mean age of 63.2 years±10.7. The blood samples were collected at the time of diagnosis, before treatment, and included 33 squamous cell carcinomas (SCC), 46 adenocarcinomas, 11 undifferentiated NSCLC, 1 large cells and 1 mixed carcinomas, of which 77,0% were male and 75,8% smokers or former smokers, divided in 42 non-metastatic and 50 metastatic cases (Table 1).

**Table 1:** NSCLC patients' characteristics.

	Patients(n = 92)	
	n	%
**Gender**		
Female	21	22.8
Male	71	77.2
**Age**		
Mean±S.D.	63.2±10.7	
**Histology**		
Adenocarcinoma	46	50.0
SCC	33	35.8
NSCLC NOS	11	12.0
Large CellsMixed	1	1.10
	1	1.10
**Staging**		
I	3	3.30
II	1	1.10
III	38	41.3
IV	50	54.3
**Smoking status**		
Non-smoker	22	23.9
Smoker	48	52.2
Former smoker	22	23.9

**Figure 1:**
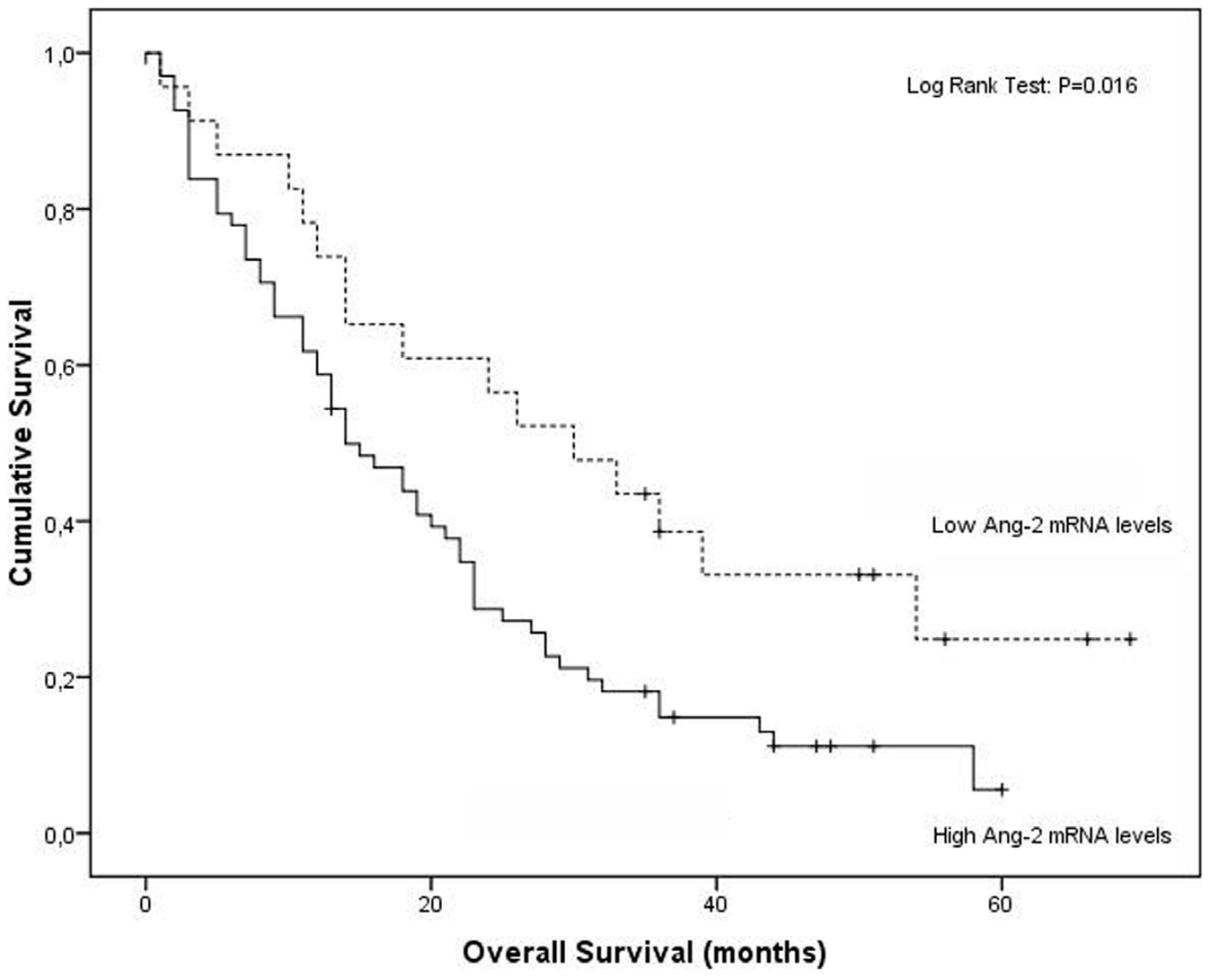
Association of high and low circulating levels of Ang-2 mRNA with overall survival in NSCLC by Kaplan-Meier curves.

**Figure 2:**
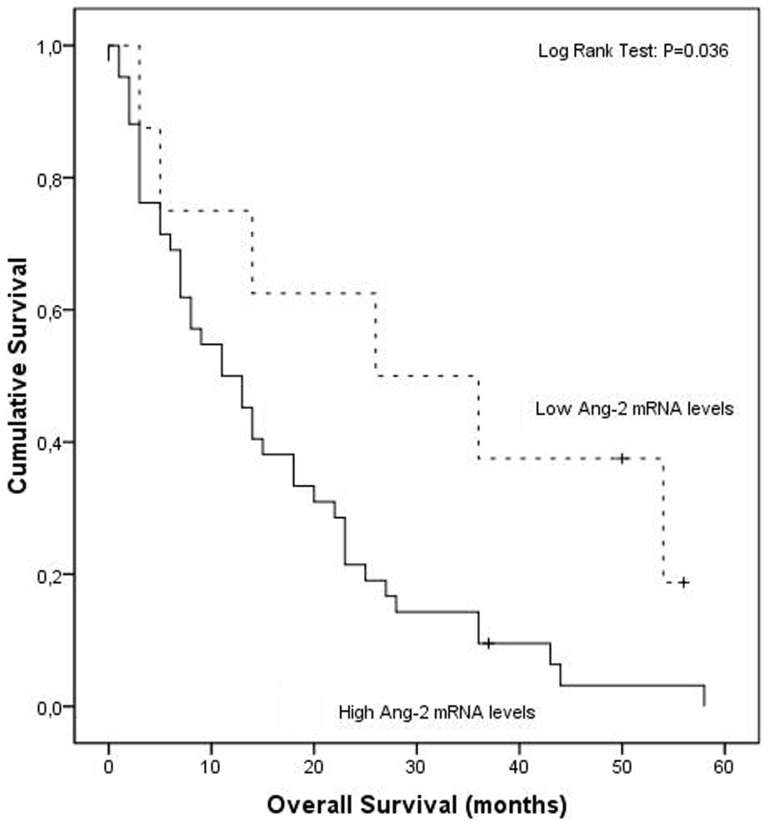
Association of high and low circulating levels of Ang-2 mRNA with overall survival in stage IV NSCLC by Kaplan-Meier curves.

Our results demonstrate that patients with high circulating Ang-2 mRNA levels have diminished overall survival when compared to those with low mRNA expression (20.3 months vs 34.3 months, respectively; Log Rank Test, p = 0.016) (Figure 1). Moreover, when considering only stage IV patients, the most suitable candidates to antiangiogenic treatment, the range interval between overall survival in the high and low settings of Ang-2 mRNA expression augments (15.9 months vs 31.3 months, respectively; Log Rank Test, p = 0.036) (Figure 2).

To determine the independent prognostic value of circulating Ang-2 mRNA levels for OS, a multivariate analysis using a Cox proportional hazard model was performed. In the multivariate analysis that included age, gender, tumor stage, histological type, smoking status and circulating Ang-2 mRNA levels, we identified tumor stage, smoking status and Ang-2 mRNA levels as independent prognostic factors for OS in NSCLC patients (Table 2).

**Table 2:** Multivariate Cox regression analysis for predictable factors of overall survival.

	HR	95% CI	P
Age (≥63;<63)	0.97	0.0.610–1.55	0.905
Gender	0.55	0.260–1.16	0.118
Tumor stage	1.79	1.12–2.88	0.016
Histological type	1.12	0.810–1.53	0.503
Smoking status	2.36	1.09–5.09	0.029
Ang-2 mRNA	2.04	1.13–3.67	0.017

Attending to these results, we performed an analysis considering four different prognostic models to ascertain the predictive power of circulating Ang-2 mRNA levels in the clinical outcome of NSCLC patients (Table 3). In the first two models, we considered the predictive ability of tumor stage and smoking status (c-index 0.657 for tumor stage and 0.522 for smoking status) (Model 1 and Model 2). Model 3 addressed the question whether circulating Ang-2 mRNA levels could also be considered a prognostic factor in NSCLC, with a c-index of 0.629 (Model 3). In model 4, we created a three variables prognostic nomogram congregating the three aforementioned models. The prognostic predictive ability was increased when adding circulating Ang-2 mRNA levels, with a c-index of 0.798 (Model 4).

**Table 3:** Predictive models of OS according to independent prognostic factors.

	HR	95% CI	P	c-index
Model 1				
Tumor stage	2.30	1.85–2.86	<0.0001	0.657
Model 2				
Smoking status	1.15	0.90–1.47	0.266	0.522
Model 3				
Ang-2 mRNA	1.92	1.10–3.36	0.021	0.629
Model 4 - Nomogram				0.798
Tumor stage	1.81	1.13–2.90	0.013	
Smoking status	1.58	0.91–2.74	0.103	
Ang-2 mRNA	1.94	1.09–3.43	0.024	

## Discussion

Many predictive and prognostic markers have been assessed in NSCLC but, until the discovery of the importance of Epidermal Growth Factor Receptor gene (*EGFR*) [18], no single molecular marker had proven to be useful for either patient selection or selection of specific drugs. The TNM classification (lung cancer staging) has stood the test of time and to date, no other prognostic factors beyond it have been prospectively validated, remaining the most powerful prognostic instrument in lung cancer [19], [20]. Hence, the identification of other prognostic factors that can be integrated with TNM to create a composite prognostic index for NSCLC would be clinically useful.

Many clinical trials have demonstrated the importance of evaluating several molecular biomarkers of NSCLC tumor specimens to allow a personalized medicine, enhancing progression free survival and overall survival times, diminishing side effects, giving patients a better quality of life and enabling to perform more cost-effectiveness treatments [21]. Moreover, we now know that in addition to predictors of response, these genes can also be regarded as prognostic factors for NSCLC [21], making the evaluation of these biomarkers the state of the art of the advanced or metastatic NSCLC treatment. However, only patients with lung adenocarcinoma with *EGFR* mutations or anaplastic lymphoma kinase (*ALK*) rearrangements have an FDA-approved therapy available [18], [21]–[23]. Unfortunately, for squamous cell NSCLC the scenario is even more shadowy. Although this is an important field where novel targeted therapies are currently under investigation, the disease prognosis still remains disappointing.

As the field of lung cancer moves further into the age of personalized medicine, alternative targets continue to be investigated, since it has become clear that it will be imperative to target the tumor stroma and surrounding environment and not merely the genes' mutations within the cancer cells itself. The main goal of this quest is to identify a pan-NSCLC prognostic factor that can also help to predict treatment response and monitor tumor progression. One of the most extensive studied of such alternative targets is angiogenesis, a necessary process in the growth and metastasis of all solid tumors [23]. Preclinical models and selected clinical trials showed benefits for targeting angiogenesis in lung cancer, with antiangiogenic treatment emerging as the first effective anti-stroma therapy to complement the established antitumor therapies [7]. So far, only VEGF, the master switch of the angiogenic cascade, has been validated as a therapeutic target for antiangiogenic intervention [7] but no published clinical study has proved that circulating levels of this target are prognostic factors in patients with NSCLC subjected to antiangiogenic therapy [24].

Despite of the advances into the elucidation of the tumor milieu in general and tumor angiogenesis in particular, there is a significant knowledge deficit in the understanding of the molecular basis of antiangiogenic therapy and the related adverse events seen with these agents [23]. Researchers have been searching for potential biomarkers to identify patients for whom therapy with antiangiogenic inhibitors may be most beneficial and the importance of these as prognostic factors in NSCLC.

Whereas VEGF is abundantly expressed by the tumor cells in most tumors, Ang-2 is mostly expressed by the tumor-associated endothelium [7]. Moreover, unlike VEGF, Ang-2 shows limited postnatal expression in normal tissues and its broad expression and prominent upregulation in tumor milieu turns it in the perfect candidate to help to define prognosis in solid tumors, besides being a suitable suspect in the game of antiangiogenic strategies [7], [25].

In the present study, we aimed to evaluate the prognostic significance of Ang-2 mRNA detection in the cell fraction of peripheral blood of patients with NSCLC prior to treatment, using qRT-PCR. Moreover, we wanted to assess the possibility of using it as a prognostic factor that could be adjoined to NSCLC staging to create a composite prognostic index for NSCLC and created a nomogram that predicts the influence of circulating Ang-2 mRNA levels in NSCLC clinical outcome.

Our results demonstrate that high circulating Ang-2 mRNA levels are a significantly unfavorable prognostic factor in NSCLC overall survival. Patients with high circulating Ang-2 mRNA levels have diminished overall survival when compared to those with low mRNA expression, when considering all NSCLC stages and this difference is even bigger when considering only patients with distant metastasis, the most suitable candidates to antiangiogenic therapies. Moreover, mRNA levels independently determine survival, and its prognostic predictive ability increases when modeled in a simple and easy to apply nomogram with NSCLC staging, patients' smoking status and Ang-2 mRNA levels (c-index 657 vs c-index 0.798, respectively).

Taken together, these results prompt us to think that detection and quantification of circulating Ang-2 mRNA in blood samples, along with proper NSCLC staging, could serve as a unique and practical diagnostic tool to determine prognosis in NSCLC. Circulating Ang-2 mRNA levels samples are a simple to obtain factor which can theoretically reflect the overall angiogenic activity of the tumor and offers a huge advantage over tissue based markers, including the ability to carry out continuous, noninvasive assessments over time and most important, not relying on the availability of adequate surgical or biopsy specimens of NSCLC.

Various therapeutic agents targeting Ang-2 have been described and are being evaluated in early-phase clinical trials [5], [9], [25]–[28]. Albeit antiangiogenic drugs are efficacious in unselected populations, increasing market competition between targeted therapies is likely to drive the growth of individualized chemotherapy, with a central role for biomarkers. Although more studies are needed to confirm this hypothesis, the circulating Ang-2 mRNA levels could be strong candidates for predicting the survival benefit associated with the targeted therapies currently under evaluation and should be included in the design of preclinical studies and clinical trials involving antiangiogenic drugs targeting Ang-2, to guide adequate patient stratification and dose selection and increasing the likelihood of benefit to a level that is acceptable to patients and clinicians.
