# Correction: Circulating Ang-2 Mrna Expression Levels: Looking ahead to a New Prognostic Factor for NSCLC

**DOI:** 10.1371/journal.pone.0097979

**Published:** 2014-05-12

**Authors:** The PLOS ONE Staff

## Notice of Republication

This article was republished on April 25, 2014, to correct errors in the title that were introduced during the typesetting process. The correct title of the article is “Circulating Ang-2 mRNA Expression Levels: Looking Ahead to a New Prognostic Factor for NSCLC.” Please download this article again to view the correct version. The originally published, uncorrected article and the republished, corrected article are provided here for reference.

## Supporting Information

File S1 Originally published, uncorrected article.
(PDF)

File S2 Republished corrected article.
(PDF)
